# Pregnancy Stress Exposures and Postpartum Serum Metabolomic Profiles in Mothers

**DOI:** 10.3390/metabo16050312

**Published:** 2026-05-01

**Authors:** Katherine Svensson, Sandra India-Aldana, Hachem Saddiki, Lauren M. Petrick, Haibin Guan, Carmen Hernández-Chávez, Martha M. Téllez-Rojo, Robert O. Wright, Rosalind J. Wright, Elena Colicino

**Affiliations:** 1 Department of Environmental Medicine, Icahn School of Medicine at Mount Sinai, New York, NY 10029, USAelena.colicino@mssm.edu (E.C.); 2 Department of Developmental Neurobiology, National Institute of Perinatology, Mexico City 11000, Mexico; 3 Center for Nutrition and Health Research, National Institute of Public Health, Cuernavaca 62100, Mexico; 4 Department of Pediatrics, Icahn School of Medicine at Mount Sinai, New York, NY 10029, USA; 5 Department of Biosciences, University of Milan, 20133 Milan, Italy

**Keywords:** metabolomics, psychosocial stress, pregnancy, women

## Abstract

Background/Objectives: Women exposed to high psychosocial stress in pregnancy have higher risk of postpartum health conditions, but it still is unknown whether high pregnancy stress exposure alters the maternal metabolome at one-month postpartum. Methods: We analyzed data from 625 women participating in the PROGRESS study, a longitudinal pregnancy cohort. Women answered validated psychometric tests (i.e., EPDS, PSS and NLE) during the second or third trimester of pregnancy and provided serum samples at one-month postpartum for metabolomic assessment. Untargeted metabolomics were analyzed using chromatography high-resolution mass spectrometry (LC-HRMS). We used a metabolome-wide association study, using both traditional robust regressions and variance tests, to evaluate associations between pregnancy psychosocial stress and one-month postpartum serum metabolomics. Results: We found a few nominally significant associations between prenatal psychosocial stress scores and the maternal metabolome. However, these findings did not remain after adjusting for multiple testing, with the only exception of epiandrosterone glucuronide, a steroid hormone metabolite, and lithocholyltaurine, a lipid-like molecule. Conclusions: We did not find significant associations between prenatal psychosocial stress and postpartum serum metabolomic profiles, except for two metabolites showing suggestive associations warranting further investigation.

## 1. Introduction

High levels of psychological stress exposure during pregnancy have been linked to increased risk of postpartum conditions in women, such as depression, anxiety, and even suicidal events. Also, epidemiological studies show that women who experience a high burden of stress and have low social support may be at higher risk for mental health conditions when going through the physiological and emotional changes occurring in prenatal and postnatal periods [1,2,3]. It is key to identify early biomarkers of prior stressful exposures and help women during and after pregnancy with potential targeted interventions. There is limited epidemiological research showing that stress exposure during pregnancy may contribute to concurrent changes in the maternal metabolome [4,5,6]. Also, animal studies support those associations, showing that rats exposed to highly stressful situations have an altered metabolome as compared to their less stressed counterparts [7,8,9,10]. However, little is known about whether psychosocial stress exposure during pregnancy is prospectively associated with one-month postpartum metabolomics data. Therefore, it is important to evaluate if the postpartum maternal metabolome is sensitive to exposure to psychosocial stress during pregnancy.

The traditional statistical approach to evaluate the associations between exposures and continuous high-dimensional metabolomics data leverages metabolomic wide association studies (MWASs), which identify differences in the central tendency (mean or median) by level of exposure. Medians are widely used for high-dimensional outcome data, due to limited ability to capture outliers in the high-dimensional setting. However, an alternative approach consists of evaluating the biomarker variance [11,12], which has been linked to different disease phenotypes [13]. This statistical approach has yet to be applied in the field of metabolomics, although sources of variability across populations have been suggested. Variability in the human metabolome may be explained by individual characteristics, such as BMI, age, and nutrition, but also environmental factors [14,15]. Early signs of diseases reflect prior exposures and may not be limited to the difference in the central tendency metabolite concentrations, but also in their variance, and may assist in identifying subgroups with different levels of susceptibility [16,17]. The mean of a biomarker may be similar between subgroups of a population while the variance differs [12]. In such scenarios, additional methods analyzing differential variability is of importance, and may help detect effect modifiers of the human metabolome [11].

The objective of this prospective cross-sectional study was to evaluate maternal stress exposure during pregnancy and the differential median and variability of one-month postpartum serum metabolites among mothers enrolled in the Programming Research in Obesity, GRowth, Environment and Social Stressors (PROGRESS) cohort.

## 2. Materials and Methods

Study population: Healthy pregnant women (18+ years of age) living in Mexico City were invited to participate in the PROGRESS cohort from 2007 to 2011 if they were at less than 20 weeks of gestation [18]. A total of 625 women underwent stress questionnaires during pregnancy and provided serum samples for metabolomics assessment at one-month postpartum. All participants had complete information on selected confounders and covariates, except for five participants who had missing information on second-hand smoking exposure and were removed from the analyses. All participants provided written consent before their participation in the study. The study protocol was approved by the institutional review boards at the Icahn School of Medicine at Mount Sinai, the Harvard T.H Chan School of Public Health, the National Institute of Public Health in Mexico and the National Institute of Perinatology. The study was performed in accordance with the Declaration of Helsinki.

Stress scores: Psychosocial stress was evaluated in multiple domains of life stress (perceived stress and negative life events) and depressive symptoms. During their 2nd and 3rd trimester of pregnancy, women answered validated psychometric questionnaires, including the 64-item Crisis in Family Systems–Revised (CRISYS) survey, the 10-item Edinburg Postnatal Depression Scale (EPDS), and the 4-item Perceived Stress Scale (PSS), which have all been validated in Spanish [19,20,21]. The CRISYS questionnaire assesses the occurrence of a series of life events during the last six months [20]. The mothers reported if an event was experienced as positive, negative or neutral. These life events are assessed across 11 domains: financial, legal, career, relationships, community and home violence, medical problems, other home issues, discrimination/prejudice, and difficulty with authority. The results generate an additive score, a negative life events (NLEs) domain score, that ranges from 0 to 11 and summarizes chronic stress through a cumulative series of negative life events across 11 domains [22]. The PSS assesses global chronic stress and establishes the degree to which respondents have experienced their lives to be unpredictable, uncontrollable, and overwhelming with regard to their coping resources over the last month [23]. Finally, the EPDS is a validated screening tool for perinatal depressive symptoms over the last seven days [19,24]. The women were asked to grade the severity and frequency of each item on four-point Likert scale. The final score ranges from 0 to 30. For each of the scales, a higher score is indicative of higher stress or more depressive symptoms. To facilitate the interpretation of findings and to enhance the contrasts, we categorized each scale by high vs. low stress levels. For EPDS we used the clinically validated score of >13, which is indicative of probable depression [24]. Previous studies have suggested a lower cut-off level for EPDS among Hispanic populations, and therefore we used the cut-off levels of 12 and 10 in sensitivity analyses [25,26]. For PSS and NLE we utilized a data-driven cut-off level classifying the higher-stress groups based on the corresponding 3rd quartile (PSS > 7 and NLE > 5). This cut-off level has previously been used to classify women with high vs. low stress during pregnancy [27].

Metabolomic data: Serum samples were collected in fasting at one-month postpartum (median (IQR): 34 (4) days) and stored frozen at −80 °C until analyzed. Untargeted metabolomics profiling was conducted, and a more detailed description of the procedure has previously been published [28]. Briefly, both polar and semi-/non-polar metabolites were identified using two analytical modes of liquid chromatography high-resolution mass spectrometry (LC-HRMS): zwitterionic hydrophilic interaction liquid chromatography (HILIC) in positive mode (ZHP), and reverse phase liquid chromatography in negative mode (RPN) [29,30]. The data was processed and normalized with established criteria. We applied SNR filtering where only metabolites with a signal-to-noise ratio of 2 median fold change in samples compared to the solvent blank were maintained. We controlled for batch effect using a Quality Control-Robust Spline Correction (QC-RSC in R library(pop)) [31]. Also, we applied a quality control criterion of 30% relative standard deviation (RSD) in repeated pooled QC injections to exclude metabolites with high technical variability. Metabolites with detected values in at least 50% of the samples were selected for analysis. Missing values were imputed with the minimum value per feature divided by √(2) and log_2_ transformed to correct for skewness. In total, 106 polar and 149 semi-/non-polar annotated metabolites were identified by matching to an in-house library considering retention time, accurate mass, and MS/MS matching (when available) with pure standards analyzed under the same conditions. This provided a high confidence level of 1 or 2 based on the Metabolomics Standards Initiative criteria [32,33]. The Personal Chemical Database Library and Profinder version B.08.00 Service Pack 3 (Agilent Technologies, Santa Clara, CA, USA) were used to identify and integrate peaks. We removed 18 xenobiotics and exogenous toxicants, which were metabolites of endocrine disrupting chemicals, resulting in 101 polar and 136 semi-/non-polar annotated metabolites (a total of n = 237 metabolites). All the processing steps of the metabolomics data were applied in the full sample of women with available metabolomics data (n = 661) before any subsequent statistical analysis of exposures or health outcomes.

Covariates: During the enrollment visit at the 2nd trimester of pregnancy, sociodemographic information was collected through questionnaires. The women’s ages were calculated based on their date of birth and date of visit. Level of education was collected in years and categorized in three levels for analysis (i.e., less than high school, high school and more than high school), and parity was dichotomized as nulliparous vs. multiparous. Women were classified as exposed to second-hand smoking if they reported that anyone in the household smoked inside the home. Pre-pregnancy body mass index (BMI) was estimated based on weight trajectories obtained from medical records and 2nd and 3rd trimester study visits [34]. Mode of delivery was obtained through medical records and categorized as vaginal or c-section (elective or scheduled). The time of blood sampling was calculated as the number of days after delivery based on the date of delivery and the date at the one-month postpartum study visit.

Statistical analysis: We first conducted a metabolome-wide study, employing robust regression models to evaluate the association between pregnancy psychosocial stress levels (high vs. low) and median levels of maternal metabolome assessed at one-month postpartum. Then we performed Brown–Forsythe variance tests (JLST R-package) [11] to determine the associations between stress levels and the variation of 237 maternal metabolites. The Brown–Forsythe variance test is less sensitive to non-normality and influential outliers and provides a more robust result as compared to other variance tests [12]. As we wanted to adjust the variance test for covariates, we used a regression-based approach to evaluate the variability between women with low and high stress levels. Therefore, the 95% confidence interval is the difference in average absolute deviations from the stress exposure group median (i.e., high vs. low stress score), adjusting for covariates. For this analysis we calculated the minimum detectable effect size with G-Power 3.1 software to achieve 80% statistical power with an α = 0.0002 (0.05 type 1 error/237 metabolites) and 625 participants. Adjusting for 7 covariates, we achieved a minimum effect size (Cohen’s d) of 0.034 for the analysis on individual metabolites with each psychosocial stress scale [35]. All analyses, including robust regression and variance analysis, were adjusted for maternal age, education, second-hand smoking, pre-pregnancy BMI, parity, mode of delivery and time of sampling. We also conducted analysis adjusting for a sparser number of covariates (i.e., maternal age, education, and second-hand smoking) with very similar results. All models were corrected for multiple testing using the false discovery rate (FDR) and statistical significance was evaluated based on a 10% FDR-adjusted *p*-value. We considered a 10% FDR threshold to identify statistical significance as previously used in other studies [36,37], and due to the exploratory nature of the study. Finally, we performed class- and pathway-based enrichment analysis as an exploratory analytical step for biological interpretation of metabolites that were nominally significantly (*p*-value < 0.05 before FDR adjustment) associated with prenatal psychosocial stress as indicated by selected psychosocial stress scales (i.e., EPDS, PSS and NLE). We used MetaboAnalyst software version 6.037, which uses six existing metabolite libraries derived from human data to provide functional and biological context to metabolomics data. The software provides enrichment ratio, which represents the number of hits within a particular metabolic pathway divided by the expected number of hits. The software also provides *p*-values and FDR-adjusted *p*-values for all results. The enriched pathways with FDR-adjusted *p*-values < 0.10 were considered significant and utilized for further biological interpretation.

## 3. Results

Women were (mean ± standard deviation) 27.8 ± 5.5 years of age and most of them (41.0%) had less than a high school degree. One third of the women (30.9%) were exposed to second-hand smoking during pregnancy. Over one fifth of the women had high scores on the EPDS (21%) and PSS (22%), while 15% of women had a high NLE score (Table 1). There were a few differences in sociodemographic characteristics across the low and high levels of psychosocial stress. These were specifically differences in age across low and high PSS score, education level across low and high EPDS and PSS scores, and parity across low and high EPDS and NLE scores.

**Figure 1:**
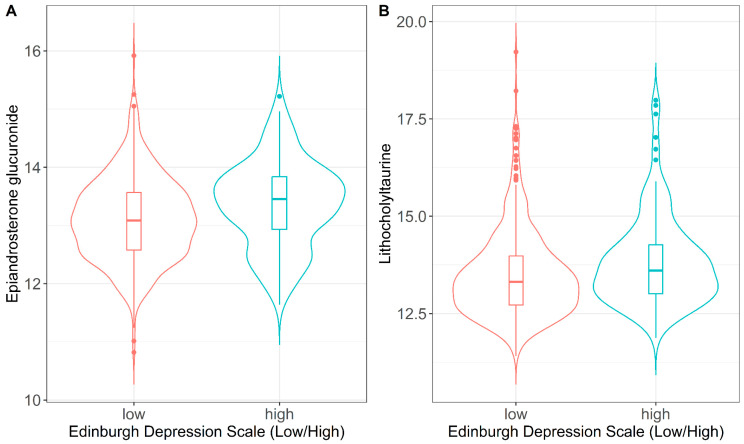
Bean plots showing the distribution of epiandrosterone glucuronide (**A**) and lithocholyltaurine (**B**) showing the median and variance among women with low vs. high EPDS (Edinburgh Postnatal Depression Scale) scores.

The metabolome-wide association study identified 27 maternal metabolites nominally associated (*p*-value < 0.05 before FDR adjustment) with at least one metric of pregnancy stress exposure (Figure S1; Table S1). After correcting for multiple testing comparisons, epiandrosterone glucuronide and lithocholyltaurine were the only metabolites showing higher median levels among women exposed to high EPDS score (β = 0.25; 95%CI: 0.11–0.40; FDR adjusted-value = 0.041; β = 0.34; 95%CI: 0.15–0.53; FDR adjusted-value = 0.041, respectively) compared to those with low scores (Figure 1; Table S1). We illustrate the median and variance of epiandrosterone glucuronide and lithocholyltaurine across high and low stress groups using bean plots (Figure 1). There were metabolites that were nominally associated (*p*-value < 0.05 before FDR adjustment) with more than one stress scale; hippuric acid was associated with both EPDS and PSS, and ethylmalonic acid was associated with both EPDS and NLE. However, these associations did not remain significant after adjusting for multiple testing (Tables S1 and S2).

We also ran sensitivity analysis using cut-off levels on the EPDS of 12 and 10. The results confirmed the metabolites identified in the main analysis as most of them were identified in the sensitivity analyses as well. Also, additional metabolites were nominally significant (*p*-value < 0.05 before FDR adjustment) with the EPDS but with little overlap between analyses (Tables S3 and S4).

Brown–Forsythe variance tests assessed the differential variability of each metabolite in relation to pregnancy stress levels and identified 19 metabolites nominally associated with at least one exposure metric. However, these variance differences were not significant after correcting for multiple testing comparisons (FDR-adjusted *p*-value > 0.10) (Table S2).

**Figure 2:**
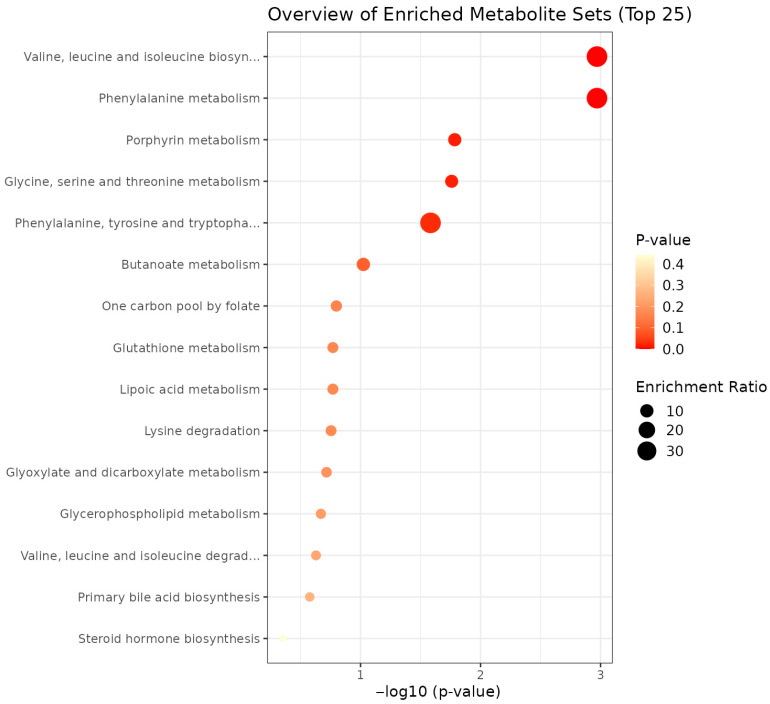
Bubble plot showing enrichment pathways for metabolites associated with prenatal psychosocial stress when analyzed using robust regressions. The X-axis denotes the −log_10_ transformed *p*-value and a darker red color denotes a higher statistical significance (i.e., lower *p*-value). The size of the bubble indicates a higher enrichment ratio. The enriched pathway analysis was conducted in MetaboAnalyst 6.0 and based on KEGG human metabolic pathways.

Pathway analysis was assessed for the metabolites that were nominally significantly (*p*-value < 0.05 before FDR adjustment) associated with prenatal psychosocial stress in the robust regressions. This step was executed purely as an exploratory analysis as only two metabolites reached significance after the FDR correction in the robust regression. The results identified enrichment of pathways in valine, leucine and isoleucine biosynthesis and phenylalanine metabolism (FDR-adjusted *p*-value < 0.10) (Figure 2). The metabolites that showed hits in these pathways were L-threonine, 3-Methyl-2-oxovaleric acid, phenylpyruvic acid, and 2-hydroxyphenylacetic acid. A second exploratory pathway analysis was also conducted after the variance test on the metabolites showing nominal significance (*p*-value < 0.05 before FDR adjustment) with psychosocial stress. The result did not show significant enrichment of pathways (FDR-adjusted *p*-value > 0.10) (Figure S2).

## 4. Discussion

Our analyses did not show significant associations between pregnancy psychosocial stress exposures and one-month postpartum metabolomics in Hispanic mothers. We only found suggested associations between EPDS score and two metabolites: epiandrosterone glucuronide and lithocholyltaurine. The epiandrosterone glucuronide is a lipid-like glucuronidated metabolite of the steroid hormone epiandrosterone. Previous research shows that changes in steroid and androgen levels are correlated with postpartum stress markers [27,38]. We used two approaches to evaluate associations, linking the exposures to both the differential median expression and differential variance expression of each metabolite. However, we did not find any differential variances between psychosocial stress scales and maternal postpartum metabolomes. Differences in group variance may bring insight into how environmental exposures may influence the spread of a metabolite before differences in the central tendency are noticed and may be indicative of population adaptation to the level of stress [16,17]. From our null findings, it may be implied that prenatal exposure to psychosocial stress does not influence the spread and the tails of the metabolite distribution in the study sample. A possible reason for the lack of significance may be due to the homogeneous population of women who were healthy at the time of enrollment without chronic diseases and relatively young, which may have limited the ability to find interindividual differences in metabolomic levels. Future studies may want to evaluate a more heterogenous population.

Our objective was to identify metabolites during the postpartum period that may be reflective of prenatal stress exposure, as has been shown in animal studies [10]. Our hypothesis was based on previous research studies showing that women with postpartum depression (PPD) have altered metabolome profiles as compared to healthy women [5,6]. In addition, previous research suggests that women with depressive symptoms may have higher levels of glutathione-disulfide, adenylosuccinate, and ATP, which are all linked to oxidative stress pathways [6]. A Canadian cohort study showed that five plasma metabolites (alanine, leucine, lactate, glucose, and phenylalanine) were at increased levels among women with depressive symptoms in late pregnancy (28–32 weeks gestation), compared with age-matched healthy pregnant controls [4]. The authors suggested a hypothesis of altered metabolism and energetic demands (i.e., mitochondrial disfunction, oxidative stress, inflammation) related to psychosocial stress [4]. However, as our results did not survive FDR correction, except for two suggested associations, we cannot make any strong conclusions or further biological interpretations of these findings. Future studies are needed to evaluate the association between prenatal psychosocial stress and women’s postpartum metabolomic profiles.

Our study uses data from the PROGRESS cohort following women from pregnancy. It provides the unique opportunity to evaluate women with high levels of psychosocial stress. In fact, relevant research focusing on psychosocial stress among adult women in Mexico City showed that one in every five pregnant women experiences symptoms of depression (prevalence of 20.5%) [39], and another study found even higher prevalence of depressive symptoms (33.5%) and perceived stress (52%) among pregnant adolescents [40]. Our study evaluated the potential to identify metabolites that could reflect high prenatal stress exposure to help identify groups at risk and who may benefit from additional support and follow-up during the postpartum period. However, we only found weak associations between two postpartum metabolites and depressive symptoms during pregnancy.

This study has some strengths and limitations. One of its strengths is leveraging the longitudinal data of the PROGRESS cohort following women over time, enabling us to analyze associations prospectively. Our results contribute to the limited literature on the relationship between prenatal psychosocial stress and women’s postpartum metabolome. Even though our results were not significant, it is a strength to have evaluated both the central tendency and the less considered variance of the metabolome levels, which may be indicative of subtle changes in population health. On the other hand, the results have limitations including the generalizability being limited to Hispanic populations, specifically Mexican women. Also, a small sample size (n = 625) in relation to the number of metabolites (n = 237) could have limited the statistical power, especially for the NLE score, where the number of women in the high stress group represented 15% of the study sample. This may have limited our ability to identify significant results and given us less precision in our estimates. We used a more lenient 10% FDR threshold for significance level due to the small sample size relative to the number of metabolites and the exploratory nature of the study. However, a more stringent FDR threshold of 5% would result in totally null findings. Therefore, we are careful to draw any strong conclusions and rather interpret our findings with epiandrosterone glucuronide and lithocholyltaurine as suggested associations. Regarding covariate adjustment, we could not adjust for diet or complications during pregnancy (e.g., preeclampsia, gestational diabetes) due to missing data. This may have limited the ability to adjust for important characteristics influencing the postpartum metabolome. In addition, the metabolome changes as women transition from pregnancy to the postpartum period [41], and even though most of the samples were collected in a one-month time window (median (IQR) of 34 (4) days), this rapid change may have made it a challenge to identify significant associations. Finally, we cannot completely rule out reverse causation, and, therefore, future studies should evaluate stress and metabolomic profiles before pregnancy.

## 5. Conclusions

In summary, our results showed non-significant associations between prenatal psychosocial stress and maternal postpartum metabolomic profiles. We found weak associations between prenatal depressive symptoms and the metabolites epiandrosterone glucuronide and lithocholyltaurine. Due to the exploratory nature of the study, our results may serve as hypothesis-generating findings and are not suitable for linking with clinical implications. In our analysis, we leveraged traditional MWAS analyses to assess the association between stress exposures and differentially expressed metabolites in a Hispanic population. We applied both robust regression analysis and variance tests to evaluate the differential variable expression of metabolites, comparing women with high vs. low stress scores (i.e., EPDS, PSS and NLE). Future studies are warranted to confirm these null findings.

## Figures and Tables

**Table 1:** Sociodemographic characteristics and self-reported psychosocial stress measures during pregnancy, n = 625.

	Overall	EPDS Low	EPDS High	PSS Low	PSS High	NLE Low	NLE High
Sociodemographic Characteristics:	Mean (SD)	Mean (SD)	Mean (SD)	Mean (SD)	Mean (SD)	Mean (SD)	Mean (SD)
Age (years)	27.8 (5.5)	27.7 (5.5)	27.82 (5.5)	28.1 (5.5)	26.7 (5.2) *	27.8 (5.6)	28.0 (5.0)
Pre-pregnancy BMI (kg/m^2^)	26.3 (4.1)	26.2 (4.2)	26.5 (4.0)	26.3 (4.2)	26.3 (4.0)	26.2 (4.2)	26.7 (4.0)
Time at blood sampling (days)	34.7 (3.7)	34.6 (3.6)	34.9 (4.1)	34.7 (3.7)	34.7 (3.7)	34.6 (3.6)	35.1 (4.1)
Education (n, %)							
Less than high school	256 (41.0)	180 (70.3)	76 (29.7) *	187 (73.0)	69 (27.0) *	221 (86.3)	35 (13.7)
High school	217 (34.7)	179 (82.5)	38 (17.5)	170 (78.3)	47 (21.7)	187 (86.2)	30 (13.8)
More than high school	152 (24.3)	134 (88.1)	18 (11.8)	132 (86.8)	20 (13.2)	125 (82.2)	27 (17.8)
Second-hand smoking exposure (n, %)							
Yes	193 (30.9)	143 (74.1)	50 (25.9)	145 (75.1)	48 (24.9)	164 (85.0)	29 (15.0)
No	432 (69.1)	350 (81.0)	82 (19.0)	344 (79.7)	88 (20.4)	369 (85.4)	63 (14.6)
Parity (n, %)							
Nulliparous	245 (39.2)	211 (86.1)	34 (13.9) *	199 (82.2)	46 (18.8)	219 (89.4)	26 (10.6) *
Multiparous	380 (60.8)	282 (74.2)	98 (25.8)	290 (76.3)	90 (23.7)	314 (82.6)	66 (17.4)
Mode of delivery (n, %)							
Vaginal/Forceps	297 (47.5)	230 (77.4)	67 (22.6)	228 (76.8)	69 (23.2)	255 (85.9)	42 (14.1)
C-section	328 (52.5)	263 (80.2)	65 (19.8)	261 (79.6)	67 (20.4)	278 (84.8)	50 (15.2)
**Stress measures:**	**Median (IQR)**	**Median (IQR)**	**Median (IQR)**	**Median (IQR)**	**Median (IQR)**	**Median (IQR)**	**Median (IQR)**
Edinburgh Postnatal Depression Scale (EPDS)							
Overall score	8.0 (9.0)	6.0 (7.0)	16.0 (3.0)	6.0 (7.0)	15.0 (6.0)	8.0 (8.0)	11.5 (8.3)
High score (EPDS > 13) (n, %)	132 (21.1)	---	---	---	---	---	---
Low score (n, %)	493 (78.9)	---	---	---	---	---	---
Perceived Stress Scale (PSS)							
Overall score	5.0 (4.0)	4.0 (3.0)	8.0 (2.0)	4.0 (3.0)	9.0 (2.0)	5.0 (4.0)	7.0 (5.0)
High score (PSS > 7) (n, %)	136 (21.8)	---	---	---	---	---	---
Low score (n, %)	489 (78.2)	---	---	---	---	---	---
Negative Life Event (NLE)							
Overall score	3.0 (3.0)	3.0 (3.0)	4.0 (3.0)	3.0 (3.0)	4.0 (3.0)	3.0 (3.0)	7.0 (2.0)
High score (NLE > 5) (n, %)	92 (14.7)	---	---	---	---	---	---
Low score (n, %)	533 (85.3)	---	---	---	---	---	---

* *p*-value < 0.05, from Student t-test for continuous variables and Chi-square test for categorical variables to evaluate differences in sociodemographic characteristics between women with low vs. high stress (i.e., EPDS, PSS, NLE). Abbreviations: EPDS, Edinburgh Postnatal Depression Scale; PSS, Perceived Stress Scale; NLE, Negative Life Event; SD, Standard deviation; IQR, Interquartile Range.

## Data Availability

The original contributions presented in this study are included in the article/Supplementary Material. Further inquiries can be directed to the corresponding author.

## Supplementary Materials

The following supporting information can be downloaded at https://www.mdpi.com/article/10.3390/metabo16050312/s1. Table S1. Nominally significant associations (*p*-value < 0.05) * from robust regression models between stress scales and one-month postpartum metabolites. Table S2. Nominally significant associations (*p*-value < 0.05) * from differential variability models between stress scales and one-month postpartum metabolites. Figure S1. Maternal stress measures associated with metabolomics at one month postpartum. Figure S2. Bubble plot showing enrichment pathways for metabolites associated with prenatal psychosocial stress when analyzed using variance test. Table S3. Nominally significant associations (*p*-value < 0.05) from robust regression models between EPDS with different cut-off levels (EPDS > 10, EPDS > 12, EPDS > 13) and one-month postpartum metabolites. Table S4. Nominally significant associations (*p*-value < 0.05) from differential variability models EPDS with different cut-off levels (EPDS > 10, EPDS > 12, EPDS > 13) and one-month postpartum metabolites.
