# Supplementary material for: Pregnancy Stress Exposures and Postpartum Serum Metabolomic Profiles in Mothers
Source: Metabolites. 2026 May 1;16(5):312. doi: 10.3390/metabo16050312 (PMC13208716; doi:10.3390/metabo16050312)
Supplement: Supplementary file 1 [file metabolites-16-00312-s001.zip › metabolites-4223404-supplementary.pdf]

metabolites

MDPI

Supplementary Material

Table S1. Nominally significant associations (p-value < 0.05) * from robust regression models between stress scales and one-month postpartum metabolites.

Metabolite	Superclass	Analytic Mode	Adj. Beta	Std. Error	95% CI Lower Limit	95% CI Upper Limit	P-value	FDR-adjusted p-value	Exposure
Carnitine	Organic nitrogen compounds	ZHP	-0.1098	0.0497	-0.2073	-0.0123	0.0275	0.9344	EPDS
4-Hydroxybenzaldehyde	Organic oxygen compounds	ZHP	-0.2198	0.1039	-0.4234	-0.0162	0.0338	0.9344	EPDS
Hippuric acid	Benzenoids	ZHP	-0.2065	0.0978	-0.3983	-0.0148	0.0345	0.9344	EPDS
Lithocholyltaurine	Lipids / lipid-like molecules	RPN	0.3378	0.0965	0.1487	0.5269	0.0005	0.0408	EPDS
Epiandrosterone glucuronide	Lipids / lipid-like molecules	RPN	0.2523	0.0729	0.1095	0.3952	0.0006	0.0408	EPDS
9,10-DiHOME	Lipids / lipid-like molecules	RPN	0.1358	0.0628	0.0128	0.2589	0.032	0.9921	EPDS
N-Acetyl-S-(N-methylcarbamoyl)-L-cysteine	Organic acids and derivatives	RPN	0.1103	0.0527	0.0069	0.2137	0.0372	0.9921	EPDS
18:2 PE (1,2-dilinoleoyl-sn-glycero-3-phosphoethanolamine)	Lipids / lipid-like molecules	RPN	0.0922	0.0453	0.0035	0.1809	0.041	0.9921	EPDS
Allothreonine/L-Threonine	Organic acids and derivatives	ZHP	-0.1383	0.0639	-0.2636	-0.013	0.0305	0.7158	PSS
Carnitine	Organic nitrogen compounds	ZHP	-0.1034	0.0488	-0.1991	-0.0077	0.0347	0.7158	PSS
Glycine	Organic acids and derivatives	ZHP	0.1401	0.0662	0.0104	0.2699	0.0351	0.7158	PSS
4-Hydroxybenzaldehyde	Organic oxygen compounds	ZHP	-0.2095	0.1019	-0.4092	-0.0098	0.0413	0.7158	PSS
Heptadecanoic acid / Hexadecanol	Lipids / lipid-like molecules	RPN	-0.1162	0.0493	-0.2128	-0.0195	0.0189	0.9059	PSS
Hippuric acid	Benzenoids	RPN	-0.239	0.1027	-0.4403	-0.0376	0.0208	0.9059	PSS
3-Hydroxybutyric acid	Organic acids and derivatives	RPN	0.1515	0.0747	0.0052	0.2979	0.0414	0.9059	PSS
N-Acety-L-Leucine	Organic acids and derivatives	ZHP	-0.3859	0.1575	-0.6946	-0.0771	0.0151	0.6666	NLE

3-Nitro-L-Tyrosine	Organic acids and derivatives	ZHP	-0.0902	0.0439	-0.1763	-0.0042	0.0391	0.6666	NLE
Gamma-aminobutyric acid	Organic acids and derivatives	ZHP	-0.1257	0.0630	-0.2492	-0.0023	0.0463	0.6666	NLE
LysoPC (14:0)	Lipids / lipid-like molecules	RPN	-0.1706	0.0656	-0.2991	-0.0422	0.0095	0.5032	NLE
Bilirubin	Organoheterocyclic compounds	RPN	-0.2522	0.1000	-0.4482	-0.0561	0.0123	0.5032	NLE
P-Hydroxyphenylacetic acid / 2-Hydroxyphenylacetic acid	Benzenoids	RPN	-0.1578	0.0643	-0.2838	-0.0319	0.0149	0.5032	NLE
Mandelic acid	Benzenoids	RPN	-0.1683	0.0714	-0.3082	-0.0283	0.0208	0.5032	NLE
17Î²-dihydroepiandrosterone sulphate	Lipids / lipid-like molecules	RPN	-0.1818	0.0805	-0.3396	-0.0241	0.0236	0.5032	NLE
Phenylpyruvic acid	Benzenoids	RPN	-0.3259	0.1447	-0.6094	-0.0424	0.0241	0.5032	NLE
1-Linoleoylglycerol_1	Lipids / lipid-like molecules	RPN	0.1447	0.0660	0.0154	0.274	0.0285	0.5032	NLE
3-Methyl-2-Oxovaleric acid	Organic acids and derivatives	RPN	-0.178	0.0805	-0.3357	-0.0203	0.0296	0.5032	NLE
N-Formyl-L-Methionine	Organic acids and derivatives	RPN	-0.1111	0.0523	-0.2136	-0.0086	0.0351	0.5304	NLE

*A total of 27 associations were nominally significant but only one surpassed the FDR threshold of 0.10. Abbreviations: EPDS, Edinburgh Postnatal Depression Scale; PSS, Perceived Stress Scale; NLE, Negative Life Event; CI, Confidence Interval; FDR, False Discovery Rate; ZHP, zwitterionic hydrophilic interaction liquid chromatography in positive mode; RPN, reverse phase liquid chromatography in negative mode.

Table S2. Nominally significant associations (p-value < 0.05)* from differential variability models between stress scales and one-month postpartum metabolites.

**Metabolite**	**Superclass**	**Analytic Mode**	**Adj. Beta**	**Std. Error**	**95% CI Lower Limit**	**95% CI Upper Limit**	**P-value**	**FDR-adjusted p-value**	**Exposure**
PAF C-16	Lipids / lipid-like molecules	ZHP	0.0644	0.0256	0.0142	0.1146	0.0122	0.9566	EPDS
Betaine/L-Valine/Norvaline	Organic acids and derivatives	ZHP	0.0486	0.0207	0.008	0.0892	0.0192	0.9566	EPDS
4-Hydroxybenzoic acid	Benzenoids	RPN	0.1269	0.054	0.021	0.2328	0.0192	0.9609	EPDS
Ethylmalonic acid	Lipids / lipid-like molecules	RPN	-0.0704	0.0328	-0.1348	-0.0061	0.0322	0.9609	EPDS
Betaine/L-Valine/Norvaline	Organic acids and derivatives	ZHP	0.058	0.0205	0.0178	0.0982	0.0048	0.4848	PSS
N-Acetylserine	Organic acids and derivatives	ZHP	-0.2553	0.1084	-0.4678	-0.0429	0.0188	0.6801	PSS

4-Aminobenzoic acid	Benzenoids	ZHP	0.1372	0.0589	0.0217	0.2527	0.0202	0.6801	PSS
7-Methylguanine	Organohetero-cyclic compounds	ZHP	-0.2665	0.1305	-0.5223	-0.0107	0.0416	0.9892	PSS
18:2 (Cis) PC (1,2-dilinoleoyl-sn-glycero-3-phosphocholine_DLPC)	Lipids / lipid-like molecules	RPN	-0.058	0.0275	-0.112	-0.0041	0.0354	0.8798	PSS
Palmitic acid	Lipids and lipid-like molecules	RPN	0.0725	0.0352	0.0036	0.1415	0.0397	0.8798	PSS
Pyroglutamic acid	Organic acids and derivatives	ZHP	-0.1096	0.0491	-0.2058	-0.0134	0.0259	0.9564	NLE
Guanidinoacetic acid	Organic acids and derivatives	ZHP	0.1518	0.0712	0.0123	0.2913	0.0333	0.9564	NLE
Phenylpyruvic acid	Benzenoids	RPN	0.211	0.0818	0.0506	0.3713	0.0102	0.6071	NLE
LysoPC(22:0)	Lipids / lipid-like molecules	RPN	-0.064	0.0286	-0.1201	-0.0078	0.0259	0.6071	NLE
3-Hydroxyphenylacetic acid	Benzenoids	RPN	-0.128	0.0584	-0.2425	-0.0136	0.0286	0.6071	NLE
LysoPC (17:0)	Lipids and lipid-like molecules	RPN	-0.0672	0.0307	-0.1273	-0.0071	0.0288	0.6071	
Ethylmalonic acid	Lipids / lipid-like molecules	RPN	-0.078	0.037	-0.1505	-0.0056	0.0352	0.6071	NLE
p-Cresol glucuronide	Organic oxygen compounds	RPN	0.2366	0.1186	0.0042	0.469	0.0464	0.6071	NLE
Mandelic acid	Benzenoids	RPN	0.1467	0.0747	0.0003	0.2931	0.0499	0.6071	NLE

*A total of 19 variance differences were nominally significant, but none surpassed the FDR threshold of 0.10. Abbreviations: EPDS, Edinburgh Postnatal Depression Scale; PSS, Perceived Stress Scale; NLE, Negative Life Event; CI, Confidence Interval; FDR, False Discovery Rate; ZHP, zwitterionic hydrophilic interaction liquid chromatography in positive mode; RPN, reverse phase liquid chromatography in negative mode.

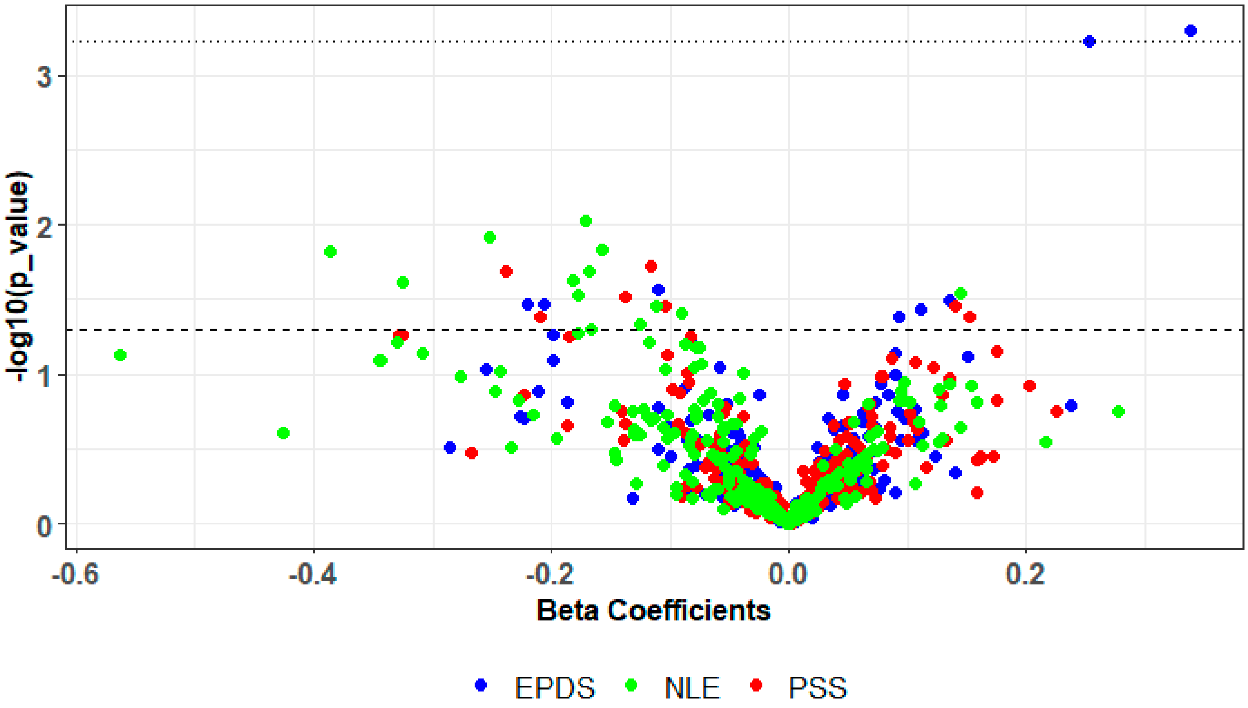

Figure S1. Maternal stress measures associated with metabolomics at one month postpartum. Volcano plots showing the adjusted beta coefficients and statistical significance (-log10 p-value) from robust regressions in women with high compared to low scores in EPDS, NLE and PSS scales and all metabolomics metabolites. All the associations were adjusted by maternal age, education, second-hand smoking, pre-pregnancy BMI, mode of delivery and time of sampling. The dots above the dashed line are nominally significant (p-value < 0.05), but only two association surpassed the FDR threshold of 0.10 (upper dotted line represents the lowest FDR-adjusted p-value in the analysis with EPDS as exposure). Abbreviations: EPDS, Edinburgh Postnatal Depression Scale; PSS, Perceived Stress Scale; NLE, Negative Life Event.

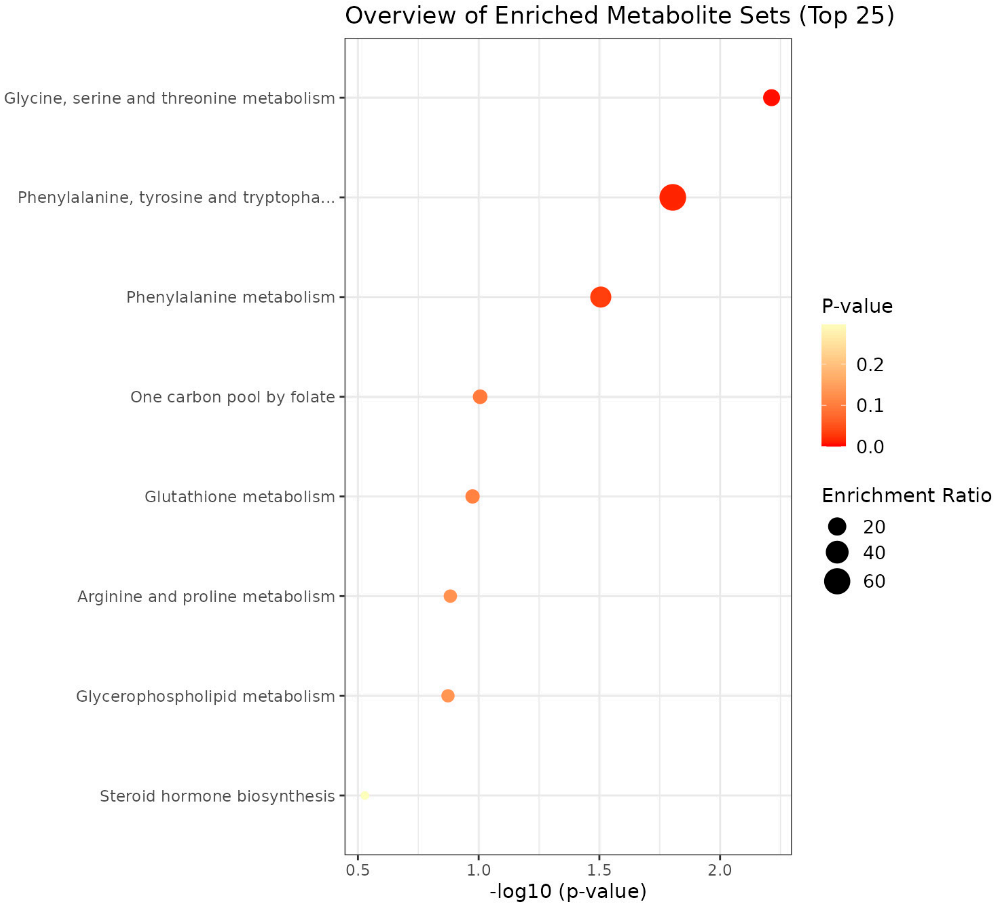

Figure S2. Bubble plot showing enrichment pathways for metabolites associated with prenatal psychosocial stress when analyzed **using variance test**. The X-axis denotes the $-\log_{10}$ transformed *p*-value and a darker red color denotes a higher statistical significance (i.e., lower *p*-value). The size of the bubble indicates a higher enrichment ratio. The enriched pathways analysis was conducted in MetaboAnalyst 6.0 based on KEGG human metabolic pathways.

Table S3. Nominally significant associations (p-value < 0.05) from robust regression models between EPDS with different cut-off levels (EPDS>10, EPDS>12, EPDS>13) and one-month postpartum metabolites.

Metabolite	Analytic Mode	Adj. Beta	Std. Error	95% CI Lower Limit	95% CI Upper Limit	P-value	FDR-adjusted p-value	Exposure
Carnitine	ZHP	-0.1098	0.0497	-0.2073	-0.0123	0.0275	0.9344	EPDS>13
4-Hydroxybenzaldehyde	ZHP	-0.2198	0.1039	-0.4234	-0.0162	0.0338	0.9344	EPDS>13
Hippuric acid	ZHP	-0.2065	0.0978	-0.3983	-0.0148	0.0345	0.9344	EPDS>13
Lithocholyltaurine	RPN	0.3378	0.0965	0.1487	0.5269	0.0005	0.0408	EPDS>13
Epiandrosterone glucuronide	RPN	0.2523	0.0729	0.1095	0.3952	0.0006	0.0408	EPDS>13
9,10-DiHOME	RPN	0.1358	0.0628	0.0128	0.2589	0.032	0.9921	EPDS>13
N-Acetyl-S-(N-methylcarbamoyl)-L-cysteine	RPN	0.1103	0.0527	0.0069	0.2137	0.0372	0.9921	EPDS>13

18:2 PE (1,2-dilinoleoyl-sn-glycero-3-phosphoethanola-mine)	RPN	0.0922	0.0453	0.0035	0.1809	0.041	0.9921	EPDS>13
Aminocaproic acid	ZHP	-0.1124	0.0402	-0.1911	-0.0336	0.0053	0.5353	EPDS>12
4-Hydroxybenzaldehyde	ZHP	-0.2287	0.0961	-0.4171	-0.0404	0.0175	0.8625	EPDS>12
Hippuric acid	ZHP	-0.1999	0.0897	-0.3758	-0.0241	0.0261	0.8625	EPDS>12
Epiandrosterone glucuronide	RPN	0.2402	0.0669	0.109	0.3713	0.0004	0.0544	EPDS>12
Lithocholyltaurine	RPN	0.296	0.0896	0.1203	0.4716	0.001	0.068	EPDS>12
Hippuric acid	RPN	-0.2308	0.0959	-0.4188	-0.0427	0.0163	0.7389	EPDS>12
9,10-DiHOME	RPN	0.1336	0.0581	0.0197	0.2474	0.0227	0.7718	EPDS>12
1-Linoleoylglycerol_2	RPN	0.1692	0.0775	0.0174	0.3211	0.029	0.7888	EPDS>12
Estriol	RPN	0.1499	0.0715	0.0099	0.29	0.0363	0.8228	EPDS>12
Homocysteine	ZHP	0.2384	0.0838	0.0742	0.4026	0.0046	0.4646	EPDS>10
N-Acetylputrescine	ZHP	0.1082	0.0426	0.0247	0.1918	0.0114	0.5757	EPDS>10
Epiandrosterone glucuronide	RPN	0.2113	0.062	0.0899	0.3328	0.0007	0.0952	EPDS>10
9,10-DiHOME	RPN	0.153	0.0537	0.0477	0.2583	0.0047	0.2221	EPDS>10
1-Linoleoylglycerol_2	RPN	0.2028	0.0718	0.0622	0.3435	0.0049	0.2221	EPDS>10
Lithocholyltaurine	RPN	0.2113	0.0836	0.0475	0.3751	0.0118	0.4012	EPDS>10
18:2 PE (1,2-dilinoleoyl-sn-glycero-3-phosphoethanola-mine)	RPN	0.0891	0.039	0.0128	0.1655	0.0225	0.5553	EPDS>10
Omega-Hydroxydodecanoic acid / 2-Undecanone	RPN	0.0995	0.046	0.0093	0.1898	0.0313	0.5553	EPDS>10
4-Hydroxybenzoic acid	RPN	-0.1399	0.0661	-0.2694	-0.0103	0.0358	0.5553	EPDS>10
Oleoyl-Glycerol	RPN	0.1749	0.0831	0.0121	0.3377	0.0358	0.5553	EPDS>10
LysoPE (18:0)	RPN	0.0873	0.0417	0.0057	0.169	0.0368	0.5553	EPDS>10
Estriol	RPN	0.1344	0.0661	0.0049	0.264	0.0428	0.5553	EPDS>10
N-Acetyl-S-(N-methylcar-bamoyl)-L-cysteine	RPN	0.0896	0.0447	0.0019	0.1772	0.0457	0.5553	EPDS>10
17Î²-dihydroepiandrosterone sulphate	RPN	0.1193	0.0606	0.0004	0.2381	0.049	0.5553	EPDS>10

Abbreviations: EPDS, Edinburgh Postnatal Depression Scale; CI, Confidence Interval; FDR, False Discovery Rate; ZHP, zwitterionic hydrophilic interaction liquid chromatography in positive mode; RPN, reverse phase liquid chromatography in negative mode.

Table S4. Nominally significant associations (p-value < 0.05) from differential variability models EPDS with different cut-off levels (EPDS>10, EPDS>12, EPDS>13) and one-month postpartum metabolites.

Metabolite	Analytic Mode	Adj. Beta	Std. Error	95% CI Lower Limit	95% CI Upper Limit	P-value	FDR-adjusted p-value	Exposure
PAF C-16	ZHP	0.0644	0.0256	0.0142	0.1146	0.0122	0.9566	EPDS>13
Betaine/L-Valine/Norvaline	ZHP	0.0486	0.0207	0.008	0.0892	0.0192	0.9566	EPDS>13
4-Hydroxybenzoic acid	RPN	0.1269	0.054	0.021	0.2328	0.0192	0.9609	EPDS>13
Ethylmalonic acid	RPN	-0.0704	0.0328	-0.1348	-0.0061	0.0322	0.9609	EPDS>13
Betaine/L-Valine/Norvaline	ZHP	0.0476	0.0189	0.0105	0.0846	0.0121	0.8336	EPDS>12
Indole-3-Acetic acid	ZHP	0.1279	0.0558	0.0186	0.2371	0.0222	0.8336	EPDS>12
4-Aminobenzoic acid	ZHP	0.1181	0.0556	0.0091	0.227	0.034	0.8336	EPDS>12
PAF C-16	ZHP	0.0479	0.0237	0.0014	0.0944	0.0441	0.8336	EPDS>12
Indole-3-Acetic acid	RPN	0.112	0.0407	0.0322	0.1918	0.0061	0.8296	EPDS>12
4-Hydroxybenzoic acid	RPN	0.1148	0.0501	0.0165	0.213	0.0224	0.9745	EPDS>12
Dehydroepiandrosterone Sulfate	RPN	0.0928	0.044	0.0066	0.1789	0.0353	0.9745	EPDS>12
Deoxycholic acid	RPN	0.2019	0.0987	0.0085	0.3953	0.0412	0.9745	EPDS>12
Linoleic acid		-0.0758	0.0378	-0.1498	-0.0017	0.0453	0.9745	
Alpha- linolenic acid / Gamma-Linolenic acid	RPN	-0.0914	0.0461	-0.1819	-0.001	0.048	0.9745	EPDS>12
Phenylacetylglutamine	ZHP	0.1782	0.0728	0.0355	0.321	0.0146	0.6985	EPDS>10
Guanosine	ZHP	-0.3144	0.1304	-0.57	-0.0587	0.0162	0.6985	EPDS>10
Indole-3-Acetic acid	ZHP	0.1193	0.0516	0.0183	0.2203	0.021	0.6985	EPDS>10
4-Aminobenzoic acid	ZHP	0.1059	0.0513	0.0054	0.2064	0.0394	0.6985	EPDS>10
Betaine/L-Valine/Norvaline	ZHP	0.035	0.0175	0.0008	0.0693	0.0454	0.6985	EPDS>10
Succinic acid	ZHP	-0.122	0.061	-0.2417	-0.0023	0.0461	0.6985	EPDS>10
4-Hydroxybenzoic acid	RPN	0.13	0.0461	0.0395	0.2204	0.005	0.5803	EPDS>10
Dehydroepiandrosterone Sulfate	RPN	0.106	0.0406	0.0264	0.1856	0.0093	0.5803	EPDS>10
Deoxycholic acid	RPN	0.2266	0.0908	0.0486	0.4046	0.0128	0.5803	
9,10-DiHOME	RPN	0.072	0.0358	0.0018	0.1423	0.0449	0.9455	EPDS>10

Abbreviations: EPDS, Edinburgh Postnatal Depression Scale; CI, Confidence Interval; FDR, False Discovery Rate; ZHP, zwitterionic hydrophilic interaction liquid chromatography in positive mode; RPN, reverse phase liquid chromatography in negative mode.
